# Roles of Type 1 Insulin-Like Growth Factor (IGF) Receptor and IGF-II in Growth Regulation: Evidence From a Patient Carrying Both an 11p Paternal Duplication and 15q Deletion

**DOI:** 10.3389/fendo.2019.00263

**Published:** 2019-04-30

**Authors:** Eloïse Giabicani, Sandra Chantot-Bastaraud, Adeline Bonnard, Myriam Rachid, Sandra Whalen, Irène Netchine, Frédéric Brioude

**Affiliations:** ^1^Sorbonne Université, INSERM, Centre de Recherche Saint Antoine, APHP, Hôpital Armand Trousseau, Explorations Fonctionnelles Endocriniennes, Paris, France; ^2^APHP, Hôpital Armand Trousseau, Département de Génétique, UF de Génétique Chromosomique, Paris, France; ^3^AP-HP, Hôpital Armand Trousseau, Department of Medical Genetics and Centre de Référence Anomalies du Développement et Syndromes Malformatifs et Déficiences Intellectuelles de Causes rares, Paris, France

**Keywords:** Beckwith-Wiedemann syndrome, IGF1 receptor, IGF-II, fetal growth restriction, imprinting disease, 11p duplication

## Abstract

We report an original association of complex genetic defects in a patient carrying both an 11p paternal duplication, resulting in the double expression of *insulin-like growth factor 2 (IGF2)*, as reported in Beckwith-Wiedemann syndrome, and a 15q terminal deletion, including the *type 1 IGF receptor gene* (*IGF1R*), resulting in haploinsufficiency for this gene. The patient was born with measurements appropriate for her gestational age but experienced growth retardation in early childhood, allowing a better comprehension of the IGF system in the pathophysiology of growth. It is possible that IGF-II plays a key role in fetal growth, independently of IGF1R signaling, and that its role is less important in post-natal growth, leaving IGF-I and growth hormone as the main actors.

## Background

**Figure 1:**
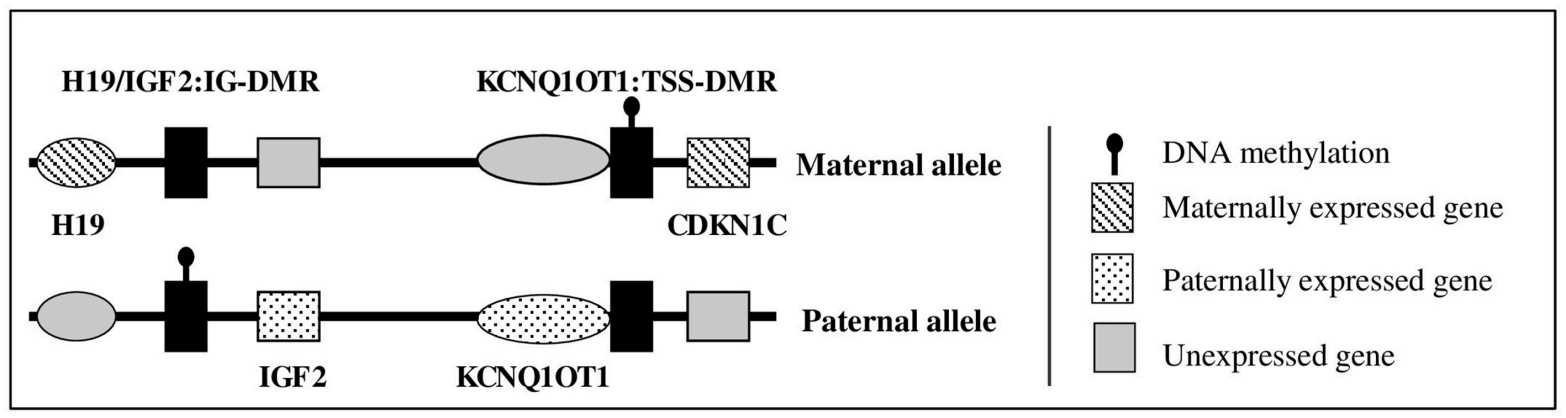
Schematic representation of 11p15 region gene expression.

Fetal and postnatal growth is a complex process, involving genetic, endocrine, and environmental factors. The insulin-like growth factor (IGF) system includes two ligands (IGF-I and IGF-II), two receptors [the IGF receptor type I (IGF1R) and the mannose-6-phosphate cation independent (M6PCI) receptor or IGF2R] (1). Circulating IGF-I is mainly produced by the liver and its production is stimulated by growth hormone (GH) after birth. *IGF2* is located in the 11p15 region in humans and is an imprinted gene expressed only from the paternal allele (Figure 1) (2). During fetal life, *IGF2* exhibits monoallelic expression and IGF-II acts as an auto/paracrine factor. After birth, circulating IGF-II is produced from both alleles by the liver, whereas *IGF2* is expressed from one allele in most other tissues and acts as a paracrine factor (3, 4). The role of circulating IGF-II after birth in humans is unclear. IGF-I and IGF-II both act through the IGF1R, which is a ubiquitously expressed tyrosine kinase receptor.

In humans, molecular anomalies of the 11p15 region have been observed in two rare diseases characterized by abnormal fetal and postnatal growth: Beckwith-Wiedemann syndrome (BWS, OMIM #130650) and Silver Russell syndrome (SRS, OMIM #180860). BWS is characterized by fetal and postnatal overgrowth, macroglossia, exomphalos, organomegaly, lateralized overgrowth, and an increased risk of embryonic tumors during early life (5). In contrast, SRS is characterized by fetal and postnatal growth retardation, with a relatively conserved head circumference at birth, hemihypotrophy, feeding difficulties, and a protruding forehead (6, 7). The 11p15 region contains two domains: the telomeric domain, containing *IGF2*, only expressed from the paternal allele, and the maternally expressed *H19* gene (a long non-coding RNA); and the centromeric domain, which includes the maternally expressed *cyclin-dependent kinase inhibitor 1C* (*CDKN1C)* gene (a negative regulator of the cell cycle, which reduces fetal growth) (Figure 1) (2, 8, 9). The expression of *H19* and *IGF2* is controlled by an imprinting center, called the *H19*/*IGF2* intergenic differentially methylated region (IG-DMR) (previously called IC1), which is methylated on the paternal allele. *CDKN1C* expression is controlled by a second imprinting center called *KCNQ1OT1*:TSS-DMR (or IC2), which is methylated on the maternal allele. Abnormal methylation of IC1 or IC2 or uniparental disomy can lead to abnormal expression of *IGF2* and/or *CDKN1C*, resulting in abnormal fetal/postnatal growth (2, 6). Duplications of 11p15 have been rarely reported, with either overgrowth or growth retardation, depending on the gene content and the parental origin of the duplication (9–11).

*IGF1R* is located on chromosome 15q26 and spans 315kb. *IGF1R* disruption (OMIM#270450) is usually responsible for fetal and postnatal growth retardation, with paradoxically high levels of plasma IGF-I (defining IGF-I resistance) and can be associated with microcephaly, variable levels of cognitive impairment, micrognathia, and feeding difficulties (12, 13). The phenotype is highly heterogeneous. In most cases, the anomaly is present in a heterozygous state, but rare homozygous or compound heterozygous mutation carriers have been reported (13–15).

We report here a patient with postnatal growth retardation and a complex chromosomal rearrangement, including a distal 15q26.3-qter deletion, encompassing the telomeric part of *IGF1R*, and a mosaic paternal duplication of the entire 11p15 region. Although the 11p duplication should have led to BWS, the patient presented with growth retardation, microcephaly, and intellectual disability, which is in accordance with the *IGF1R* disruption phenotype. We discuss the impact of these two rare genetic defects on the growth phenotype, which highlights the major role of IGF1R in IGF-II signaling.

## Case Presentation

### Clinical Aspects

**Figure 2:**
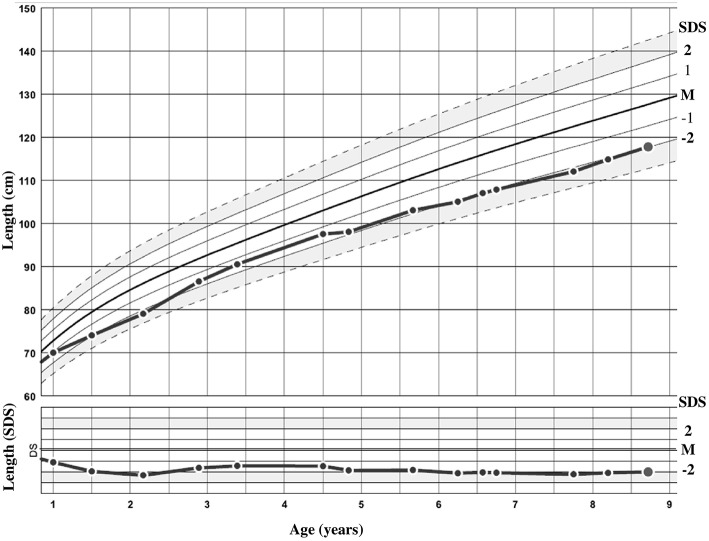
Growth curve of the patient in centimeters and standard deviation score (SDS).

The patient was sent to a reference tertiary center because of intellectual disability. She was born after 36 weeks of amenorrhea (WA), with birth parameters appropriate for gestational age (AGA). Her birth weight was 2380 g [−0.6 standard deviation score (SDS)]. Her birth length was not recorded, but at 1 month of age (equivalent to 40 WA), it was 50 cm (in the normal range). Her head circumference at birth was 30 cm (−2.4 SDS). She was born from unrelated healthy parents of Romanian origin. The mother is 162 cm (−0.2 SDS) and the father 170 cm (−0.8 SDS) tall; both had birth parameters AGA. The target height was 159.5 cm (−0.7 SDS) and there was no familial history of short stature. The growth curve is shown in Figure 2. At the age of 8 years and 9 months, her height was 117.8 cm (−2.1 SDS), weight 24.5 kg [body mass index (BMI) of 17.7 kg/m^2^ (1.2 SDS)], and head circumference 47.5 cm (−3.2 SDS). She had no clinical signs of BWS according to the consensus clinical scoring system proposed in 2018 (5). Only two out of four items for the clinical scoring system for *IGF1R* defects were present (16). She presented with strabismus and interventricular communication, with no cardiac failure. She acquired motor skills with normal timing but experienced an early delay in language and cognitive development and required specialized education.

### Biological Aspects

At the age of 3 years, her serum IGF-I level was in the upper range of the norm (145 ng/ml, 0.9 SDS), with elevated basal GH (45 mUI/L). At 8 years and 9 months, her hormonal status was as follows: IGF-I 345.3 ng/mL (2.1 SDS), IGF-binding protein (IGF-BP3) 4,638 ng/mL (normal range from 2,146 to 5,801 ng/mL), acid-labile subunit 2,145 mU/mL (normal range from 813 to 1,729 mU/mL) and IGF-II 710 ng/mL (normal range from 433 to 997 ng/mL). These data are in favor of IGF-I resistance with high levels of IGF-I and ALS.

### Molecular Aspects

**Figure 3:**
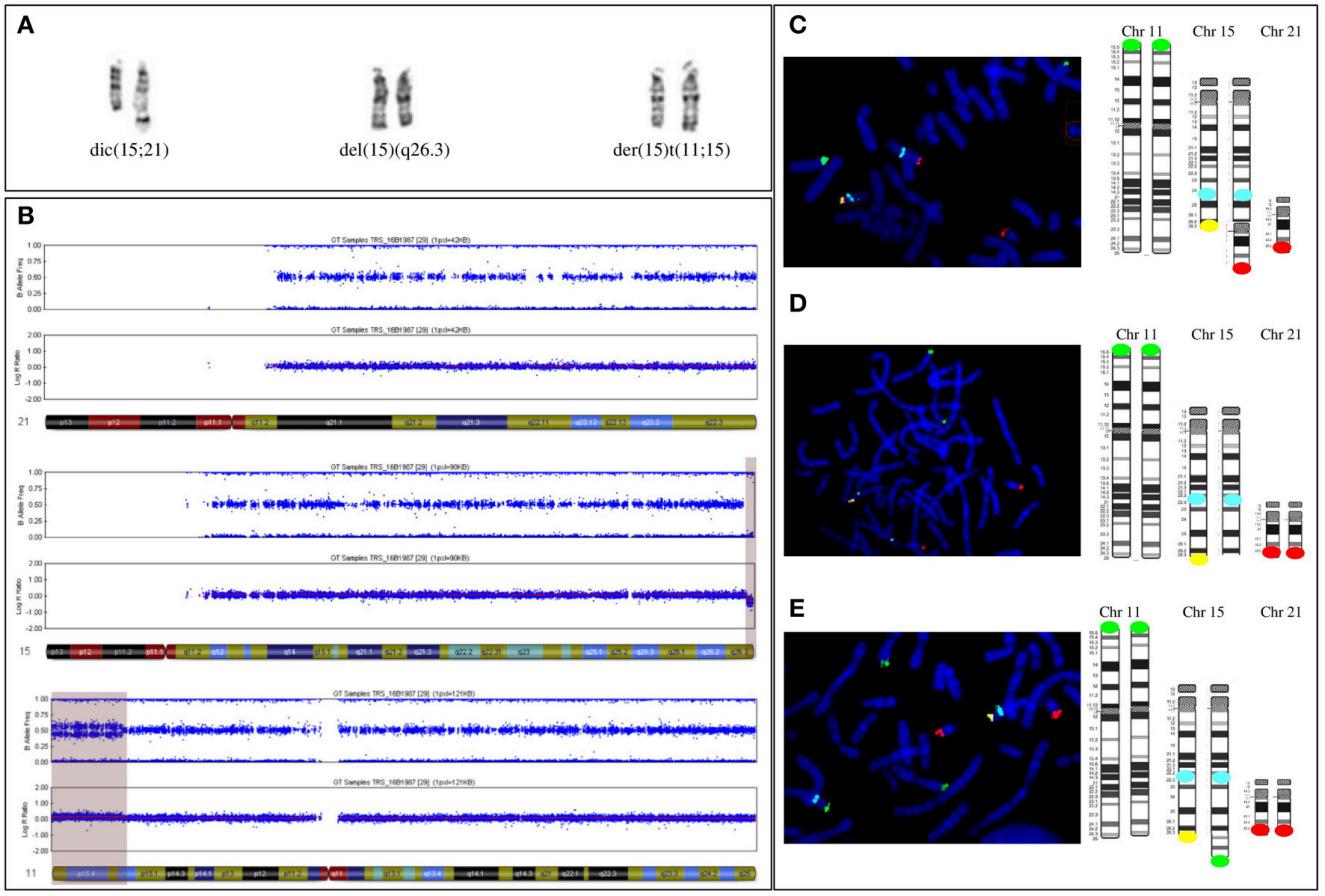
**(A)** Paired homologous chromosomes15 with Derivative chromosome 15 (left couple, on the right) and apparently normal 15 G-banded chromosomes 15 (middle couple) and (right couple). The two apparently normal chromosomes were then shown to carry a 15q terminal deletion (middle couple, on the right) and be a derivative chromosome 11 (right couple, on the right). **(B)** The SNP array profile shows a normal chromosome 21 (top), a heterozygous homogeneous 15q terminal deletion (middle, light purple area), and a mosaic gain on chromosome 11p (bottom, light purple area). **(C)** FISH [with a specific 11p subtelomeric probe (green), specific 15q22 probe (aqua blue), specific 15q subtelomeric probe (yellow), and specific subtelomeric 21q probe (red)] and schematic representation showing chromosomes 11, 15, and 21 in the three cell lines. First cell line with an unbalanced 15q;21p translocation. **(D)** Second cell line with a deleted 15q chromosome. The derivative chromosome 15 is missing a yellow signal. **(E)** Third cell line with an unbalanced 11p;15q translocation. The derivative chromosome 15 has a supernumerary green signal and no yellow signal.

Karyotype analysis revealed a mosaic karyotype 45,XX,dic(15;21)(q26.3;q10)[4]/46,XX[20] with two cell lines: (1) a 45,XX cell line with a dicentric chromosome dic(15;21) due to an apparently balanced mosaic structural rearrangement involving one chromosome 15q and one chromosome 21p (Figure 3A left) and (2) a 46,XX cell line. The karyotypes of the parents were normal.

Single-nucleotide polymorphism (SNP) array revealed a more complex unbalanced autosomal structural rearrangement than expected from the karyotype analysis: (1) no copy number variation for chromosome 21, (2) a 3 Mb homogeneous heterozygous 15qter deletion, and (3) a 13.4 Mb mosaic 11pter duplication in approximately 30% of the cells analyzed (Figure 3B from top to bottom) (10).

Subsequent fluorescent *in situ* hybridization (FISH) analysis with specific subtelomeric 11p, 15q, and 21q probes showed a supernumerary signal for 11p at the end of a deleted 15qter chromosome in ~30% of cells, accounting for an unbalanced translocation t(11p;15q), with a normal signal on the two telomeres of 21q (Figures 3A right, E). In approximately 20% of cells, one signal for the chromosome 21q telomere was located at the end of the deleted 15qter chromosome with a normal signal for the two 11p telomeric probes, accounting for an unbalanced translocation t(15q;21q) (Figures 3A left, C). In the remaining cells, with a normal signal on telomeres 11p and 21q, only one signal from the telomeric 15q probe was detected, corresponding to a 15q deletion (Figures 3A middle, D). Thus, the karyotype of the patient was finally amended to ISCN 2016 as *de novo* mos 45,XX,dic(15;21)(q26.3;q10)/46,XX.ish.der(15)(11;15)(p15.2;q26.3)(RP11-889I17+,D15S936-)/46,XX.ish.del(15)(q26.3)(D15S936-).arr[hg19]11p15.5p15.2(204,062-13,618,804)x3[0.3],15q26.3(99,434,357-102,461,162)x1dn.

Thus, the patient's mosaic included three abnormal cell lines: the first (30%) with 46 chromosomes and a chromosome 15 derived from an unbalanced translocation between the chromosome 11p region and the terminal 15q26 region of chromosome 15; the second (20%) with 45 chromosomes and an unbalanced dicentric chromosome derived from both chromosomes 21 and 15, with a 15q26 terminal deletion, and the third (50%) with 46 chromosomes that only carried the 15q26 terminal deletion of one chromosome.

Methylation studies (using methylation-specific multiplex ligation dependent probe amplification after bisulfite treatment of DNA) of the 11p15 locus revealed a partial gain of methylation of the *H19/IGF2*:IG-DMR (methylation index of 55; normal range 46 à 51) and a partial loss of methylation of the *KCNQ1OT1*:TSS-DMR (methylation index of 43; normal range 48- 53), in favor of a paternal origin of the 11p duplication (17).

## Discussion

We report a patient with a complex chromosomal rearrangement which includes both *IGF2* and the *IGF1R*. In this case, two rare conditions coexist: a mosaic 11p15 paternal duplication, which usually leads to overgrowth (BWS), and a 15qter deletion including *IGF1R*, which usually leads to fetal and post-natal growth restriction. The major role of IGF-I and IGF–II as major actors in the control of fetal growth, through their binding to the IGF1R, has been known for years. Indeed, in humans, genetic defects of *IGF1* and the *IGF1R* or alterations in *IGF2* expression, through genetic or epigenetic mechanisms (SRS) leads to fetal growth restriction (16–18). Conversely, overexpression of *IGF2* (BWS) or the *IGF1R* lead to overgrowth (19, 20).

Moreover, murine models of invalidation of these genes confirmed these observations, as knockout models for either *Igf1, Igf2*, or *Igf1r* present with growth restriction at birth (21, 22). Interestingly, the double knockout for *Igf1* and *Igf2* or *Igf2* and *Igf1r* are smaller at birth than the knockout for *Igf1r*, whereas double knockouts for *Igf1* and *Igf1r* are the same size as the *Igf1r* knockout mice. These data suggest that, conversely to IGF-I, which only interacts with the IGF1R, IGF-II may also act through other signaling pathways (22).

In humans, many findings have highlighted the role of IGF-I in stimulating postnatal growth: patients with GH deficiency or GH resistance [Laron (OMIM#262500) or Noonan (OMIM#163950) syndromes] have extremely low levels of IGF-I and present with growth failure after birth (12). Conversely, patients with acromegalogigantism (because of GH pituitary adenoma) have extremely high levels of IGF-I and tall stature.

The role of IGF-II in postnatal growth is unclear. Indeed, SRS patients with a loss of methylation at the *H19/IGF2*:IG-DMR present with persistent post-natal growth failure, despite normal circulating IGF-II levels (23, 24). Such normal circulating IGF-II levels may be due to the biallelic expression of *IGF2* from a non-imprinted promotor in the liver. However, *IGF2* is still imprinted in other tissues, and loss of methylation at 11p15 leads to the loss of *IGF2* expression in these tissues (4, 17). Thus, these plasmatic levels of IGF-II do not reflect the local levels and activity of IGF-II. 11p15.5 paternal duplications have been recently reviewed (10). In such duplications, either growth retardation (SRS), overgrowth (BWS), or a normal phenotype can be observed, depending on the extent of the duplication and the parental origin of the duplicated allele. Usually, duplications of the paternal telomeric domain of 11p15 leads to *IGF2* overexpression and thus BWS, whereas a gain of *CDKN1C* expression, due to maternal duplication of the centromeric domain of 11p15, leads to SRS (10). In our patient, we demonstrate that the duplicated 11p15 allele was of paternal origin and encompassed both IC1 and IC2. Thus, *IGF2* is likely overexpressed in the contingent of cells with the 11p15 duplication. However, in this patient plasmatic IGF-II levels were within the normal range which could be secondary to a different mosaicism in the liver with a lower rate of cells carrying the 11p15 duplication.

The growth retardation, microcephaly, developmental delay, and high plasma levels of IGF-I observed in our patient are more concordant with the *IGF1R* deletion phenotype. Here, fetal growth was not affected, despite *IGF2* overexpression and the coexisting *IGF1R* defect. These two opposite mechanisms may compensate each other and finally lead to normal birth parameters. Nevertheless, we should be cautious when speculating on the respective role of genetic anomalies impact here since we do not know the exact proportion of mosaicism in the different tissues. Information on placenta, for example, would have been of major interest since IGF-II and IGF1R play a major role on placental development and function (25). It reinforces the hypothesis that IGF-II can signal through a pathway that is independent of the IGF1R, at least during fetal life (22). Finally, *in vitro* studies have also shown a stronger affinity of IGF-I than IGF-II for IGF1R and IGF1R is activated with lower concentrations of IGF-I compared to IGF-II (26, 27).

The growth retardation our patient experienced suggests that the IGF1R defect prevails over IGF-II overexpression after birth. Indeed, post-natal growth retardation with a biological IGF-I resistance profile is concordant with the predominant dysfunction of the IGF1R. In patients with low *IGF2* expression (SRS), the fetal growth restriction is obvious, whereas their growth velocity is usually unaffected after birth (despite no catch-up) (23). Conversely a comparison of patients with BWS shows birth length to generally be greater in patients with *IGF2* overexpression (gain of methylation in IC1), whereas height during childhood is usually greater in patients with an isolated IC2 loss of methylation (no *IGF2* overexpression) (19). This favors a predominant role of IGF-II in fetal rather than in post-natal growth. For the patient reported here, another explanation for the normal measurements at birth may lie in the mosaicism presented by the patient, since all her circulating cells carried the *IGF1R* deletion, but only approximately 30% had the 11p duplication. This mosaicism may vary across various tissues, and the growth plate may have a different proportion of cells with the 11p duplication.

Patients with *IGF1* or *IGF1R* defects usually present with microcephaly, which distinguishes them from SRS patients, for whom head circumference is relatively conserved at birth. This would favor a major role of IGF-I in cerebral development over that of IGF-II (28). Another possibility is the absence of maternal imprinting of *IGF2* in brain, as several studies found either biallelic expression in different cerebral regions or, more recently a maternal expression (4, 21, 29). In the latter case, *IGF2* would not be overexpressed in the brain of the patient (because of the paternal origin of the duplication) conversely to the other tissues. Thus, *IGF1R* haploinsufficiency would have led to the microcephaly, in accordance with previous observations of *IGF1R* disruption.

## Conclusion

We report here an original association of multiple chromosomal rearrangements involving *IGF2* and *IGF1R*, two critical genes involved in the regulation of fetal and post-natal growth regulation. It favors the predominance of IGF-II during fetal growth and IGF-I during post-natal growth.

## Ethics Statement

A written informed consent was obtained from the parents of the participant for the publication of this case report.

## Author Contributions

EG, SC-B, and FB shared writing of the manuscript. AB, MR, and SC-B performed the molecular tests and analysis. SW and IN participated in the discussion and revisions during the writing process.

### Conflict of Interest Statement

The authors declare that the research was conducted in the absence of any commercial or financial relationships that could be construed as a potential conflict of interest. The reviewer TE declared a past co-authorship with the authors to the handling Editor.
